# Perceived barriers to randomised controlled trials in breast reconstruction: obstacle to trial initiation or opportunity to resolve? A qualitative study

**DOI:** 10.1186/s13063-020-4227-1

**Published:** 2020-04-06

**Authors:** Gareth Davies, Nicola Mills, Chris Holcombe, Shelley Potter, on behalf of the iBRA Steering Group, N. L. P. Barnes, J. M. Blazeby, O. A. Branford, R. I. Cutress, M. D. Gardiner, C. Holcombe, A. Jain, K. McEvoy, N. Mills, S. Mylvaganam, S. Potter, J. M. Skillman, E. M. Teasdale, S. Thrush, Z. Tolkien, L. J. Whisker

**Affiliations:** 1 Bristol Centre for Surgical Research, Population Health Sciences, Bristol Medical School, Canynge Hall, 39 Whatley Road, Bristol, BS8 2PS UK; 2 Linda McCartney Centre, Royal Liverpool and Broadgreen University Hospital, Prescot Street, Liverpool, L7 8XP UK; 3 Bristol Breast Care Centre, North Bristol NHS Trust, Southmead Road, Bristol, BS10 5NB UK

**Keywords:** Qualitative, randomised controlled trials, Surgery, Implant breast reconstruction

## Abstract

**Background:**

Implant-based breast reconstruction (IBBR) is the most commonly performed breast reconstruction technique worldwide but the technique is evolving rapidly. High-quality evidence is needed to support practice. Randomised controlled trials (RCTs) provide the best evidence but can be challenging to conduct.

iBRA is a four-phased study which aimed to inform the feasibility, design and conduct of an RCT in IBBR. In phase 3, the randomisation acceptability study, an electronic survey and qualitative interviews were conducted to explore professionals’ perceptions of future trials in IBBR. Findings from the interviews are presented here.

**Methods:**

Semi-structured qualitative interviews were undertaken with a purposive sample of 31 health professionals (HPs) who completed the survey to explore their attitudes to the feasibility of potential RCTs in more detail. All interviews were transcribed verbatim and data were analysed thematically using constant comparative techniques. Sampling, data collection and analysis were undertaken iteratively and concurrently until data saturation was achieved.

**Results:**

Almost all HPs acknowledged the need for better evidence to support the practice of IBBR and most identified RCTs as generating the highest-quality evidence. Despite highlighting potential challenges, most participants supported the need for an RCT in IBBR. A minority, however, were strongly opposed to a future trial. The opposition and challenges identified centred around three key themes; (i) limited understanding of pragmatic study design and the value of randomisation in minimising bias; (ii) clinician and patient equipoise and (iii) aspects of surgical culture and training that were not supportive of RCTs.

**Conclusion:**

There is a need for well-designed, large-scale RCTs to support the current practice of IBBR but barriers to their acceptability are evident. The perceived barriers to RCTs in breast reconstruction identified in this study are not insurmountable and have previously been overcome in other similar surgical trials. This may represent an opportunity, not only to establish the evidence base for IBBR, but also to improve engagement in RCTs in breast surgery in general to ultimately improve outcomes for patients.

**Trial registration:**

International Standard Randomised Controlled Trial Number ISRCTN37664281.

## Background

Implant-based breast reconstruction (IBBR) is the most commonly offered reconstructive technique worldwide following mastectomy for breast cancer [1, 2]. Traditionally, IBBR consisted of a two-stage procedure with initial placement of a tissue expander under the pectoralis muscle followed by sequentially filling to achieve the desired volume. A second operation is then required to replace the expander with a fixed-volume implant. Over the last decade, however, biological and synthetic meshes have been introduced. These have allowed the reconstruction to be performed in a single operation with better cosmetic outcomes, which has increased the popularity of the technique [3, 4]. Most recently, muscle-sparing techniques have been introduced in which the implant, wrapped in mesh, is placed on top rather than underneath the pectoralis muscle [5]. This may decrease post-operative pain and avoid distressing implant ‘animation’, the upwards movement of the implant seen when the pectoralis muscle contracts [5].

Despite the widespread adoption of mesh-assisted techniques, there is a paucity of high-quality evidence to support their safety or effectiveness [5–8]. Multicentre prospective studies have failed to demonstrate any difference in outcomes between single-stage direct-to-implant and two-stage techniques [9, 10] or between two-stage reconstructions with and without mesh [11]. However, a recent Dutch multicentre randomised controlled trial (RCT) demonstrated significantly increased numbers of complications when single-stage direct-to-implant procedures were compared with traditional two-stage techniques [12] despite equivalent quality-of-life outcomes [13]. A second RCT [14], did not demonstrate a difference between the two techniques. The current evidence is therefore inconclusive and supports the need for further high-quality research to guide best practice in IBBR.

Well-designed large-scale RCTs are considered the ‘gold standard’ for evaluating new healthcare interventions and provide the highest levels of evidence. Despite this, they can be very challenging with less than 50% of trials estimated to meet intended recruitment targets [15]. Barriers to recruitment in RCTs are well-described [16, 17] and may be broadly divided into factors relating to the trial design; organisational or logistical issues such as delays in trial set-up, and participant and clinician-related factors [17]. Although many recruitment issues are common to all RCTs, clinician factors may be more problematic in surgical trials as surgeons are less familiar with key aspects of trial methodology [18], in particular the concepts of equipoise [19] and randomisation and may not have participated in RCTs before [20].

Furthermore, unlike many surgical procedures that are essential to save or prolong a patient’s life, breast reconstruction is an optional intervention that is performed to improve quality of life following mastectomy [21]. Trials in breast reconstruction therefore represent specific challenges due to patient and surgeon preference for different treatment options [22] and previous RCTs have been closed prematurely due to issues with recruitment [23, 24]. Before any large-scale RCTs in IBBR can be considered, careful pre-trial work is required to engage the breast reconstruction community, identify the most acceptable and feasible trial designs, and to ensure any potential barriers to trial conduct are identified and overcome.

The iBRA study (implant Breast Reconstruction evAlutation) [25] (ISRCTN37664281) is a four-phased study to inform the feasibility, design, and conduct of a future trial in immediate IBBR. Phase 1 was a national practice questionnaire aiming to survey current practice, with phase 2 involving a multicentre prospective cohort study of patients undergoing IBBR to evaluate the clinical and patient-reported outcomes of these procedures. Phase 3 of the study used mixed-methods to investigate the acceptability of candidate trial designs generated in phase 1 [26, 27] and phase 2 [9]. This included an electronic survey and qualitative interviews, which aimed to explore the acceptability of proposed RCT designs and possible barriers to RCT conduct in IBBR, from the perspective of those who would be potential trial recruiters. Findings from the interview study are presented in this article.

## Methods

This work comprises phase 3, the Randomisation Acceptability Phase of the iBRA study (ISRCTN37664281) a multicentre prospective cohort study to inform the feasibility, design and conduct of a large-scale pragmatic RCT in IBBR. The study protocol [25] and results of phases 1 [26, 27] and 2 [9] have been published elsewhere. Full ethical approval for phase 3 was obtained from the University of Bristol Faculty of Health Sciences Research Ethics Committee (FREC) Reference 61,501. This study has been reported in accordance with the COREQ (COnsolidated criteria for REporting Qualitative research) guidance [28].

### The Randomisation Acceptability Study

Phase 3, the Randomisation Acceptability Study, comprised an online questionnaire developed in REDCap [29] by the study steering group based on the results of phases 1 and 2 of the study and qualitative interviews with questionnaire respondents to further explore issues related to RCT acceptability.

The online survey (Additional file 1) aimed to explore healthcare professionals (HP’s) views of areas of uncertainty in IBBR; the need for a future RCT, possible candidate trial designs; the feasibility of recruitment and attitudes to primary outcome selection and timing of assessment. Survey participants were asked to provide consent if they were willing to be contacted to participate in a semi-structured qualitative telephone interview to explore their views in more depth. Respondents consenting to be contacted were asked to provide an e-mail address to allow an interview to be arranged.

### Survey sampling and recruitment

The Randomisation Acceptability Survey was sent electronically to all professionals involved in phases 1 or 2 of the iBRA study and a link to the REDCap survey was also circulated to all breast and plastic surgeons and clinical nurses specialists (CNS) through the professional associations and relevant trainee research networks (Association of Breast Surgery, the British Association of Plastic, Reconstructive and Aesthetic Surgeons, the Mammary Fold Academic and Research Collaborative, and the Reconstructive Surgery Trials Network). Reminders and follow up e-mails were sent at 2 and 4 weeks to optimise response rates and participation but no incentives were offered. Findings of the Randomisation Acceptability Survey will be reported elsewhere [30].

### Interview sampling and recruitment

Survey respondents who were willing to be contacted were purposively sampled and interviewed to explore common and unusual questionnaire responses to enable a more detailed understanding of the acceptability of proposed study designs and attitudes to the feasibility of RCTs in IBBR in general. A maximum variation sampling approach was employed initially to ensure a breadth of respondent professions (breast and plastic surgeons and CNSs), gender, experience, case volume and expertise of performing differing techniques (meshes and implant placement). The participant characteristics were selected based on factors shown to impact breast reconstruction practice in the literature [31] and previous work exploring attitudes to trial acceptability in this setting [22]. Deviant cases such as professionals with particularly positive, negative or other interesting attitudes to trial feasibility, design and conduct from their questionnaire responses were included to allow emerging themes to be further explored. Targeted sampling was then utilised to identify population groups that were felt to be under-represented in the initial cohort, and to follow up emerging findings of interest.

Invitations to consenting professionals were sent in batches based on the sampling strategy described above. Non-responders were followed up with personalised e-mail reminders 2 and 4 weeks later. If no response were received, the professional was considered to have declined participation and further participants were invited until data saturation had been achieved or the pool of consenting professionals had been exhausted.

### Data collection

All interviews were conducted by telephone by a medically qualified researcher who had no prior clinical experience of breast reconstruction surgery (GD). The interviewer was trained and overseen by an experienced qualitative research methodologist (NM). A topic guide was developed, based on the literature, previous questionnaire responses, clinical expertise and input from a social scientist (Additional file 2). This provided a framework of open-ended questions to guide discussion and was iteratively modified throughout data collection to allow emerging themes to be explored.

All interviews were digitally audio-recorded, transcribed verbatim and anonymised. Reflective notes were made by the interviewer immediately after interviews to capture information regarding delivery of the responses that could be lost during transcription. To ensure transcription accuracy and accurate data capture, all original interview audio recordings were checked against interview transcripts by the interviewer (GD).

### Analysis

Data analysis began soon after commencement of data collection and was repeated throughout the study period. Transcripts were imported into the qualitative data analysis software NVivo (version 11) for optimisation of data handling and analysed thematically using the constant comparative approach of grounded theory [32]. This approach was selected to ensure the findings systematically compared and were fully grounded in the data. Interviews were analysed in small batches with codes assigned systematically to segments of text to capture the meaning of those words. These codes were subsequently grouped into emerging themes and these themes were subsequently explored in later interviews. As data collection progressed, early codes and emerging themes were adapted and revised through ongoing assimilation of data. Participants’ views were compared and contrasted to explore how factors such as gender, subspecialty, experience, and case volume influenced attitudes towards the acceptability of trials in IBBR.

GD, along with two further members of the team - an experienced social scientist (NM) and a senior clinician (SP) - met regularly to review coding and descriptive findings, agree further sampling strategies and discuss emerging themes. Data collection and analysis continued concurrently and iteratively until data saturation was achieved and no new themes emerged from the data. Each participant was only interviewed once, and the transcripts were not shared with the participants.

## Results

### Demographics

Between March and August 2018, 156 professionals including 77 consultant breast surgeons; 15 consultant plastic surgeons and 36 CNS contributed to the RCT acceptability survey and 116 completed it. Of those contributing data, 109 (70%) felt there was uncertainty regarding the best practice of IBBR. Eighty-five (55%) felt that an RCT was needed and 108 (69%) felt an RCT may be possible if the design was acceptable. Further details of the survey results will be reported elsewhere.

Of the 116 professionals completing the RCT Acceptability Survey, 56 (48%) HPs (42 oncoplastic breast surgeons [OPBS], 9 plastic surgeons, 5 breast CNSs) consented to be contacted to participate in an interview. Consenting professionals were invited to participate in batches but as the response rate to the invitation e-mails was low, all consenting professionals were eventually invited to participate over the course of the study. Overall, of the 56 professionals invited, 38 (68%) replied to the invitation e-mail and 35 agreed to be interviewed. Despite agreeing to participate, it was not possible to conduct interviews with 4 respondents (2 OPBS and 2 plastic surgeon) due to scheduling issues, leaving a total of 31 completed interviews (55% of all invited). This included 27 OPBS, 2 plastic surgeons, and 2 CNS involved in breast reconstruction. The median interview length was 24 minutes (range 7–41 minutes). Table 1 shows background details of study participants. There were more female respondents (*n* = 23) than male (*n* = 8) reflecting the relatively higher number of female breast surgeons in the speciality.

**Table 1:** Details of study participants

Identifier	Profession	Sex	Experience if consultant/CNS (years)	Total N of reconstructive procedures per year at their unit
OPBS 1	Oncoplastic breast surgeon	F	>10	11–20
OPBS 2	Oncoplastic breast surgeon	F	5–10	5–10
OPBS 3	Oncoplastic breast surgeon	F	<5	50–100
OPBS 4	Oncoplastic breast surgeon	F	5–10	25–50
OPBS 5	Oncoplastic breast surgeon	M	<5	25–50
OPBS 6	Oncoplastic breast surgeon	F	<5	25–50
OPBS 7	Oncoplastic breast surgeon	F	>10	50–100
OPBS 8	Oncoplastic breast surgeon	F	<5	11–20
OPBS 9	Oncoplastic breast surgeon	F	<5	50–100
OPBS 10	Oncoplastic breast surgeon	F	<5	25–50
OPBS 11	Oncoplastic breast surgeon	F	5–10	25–50
OPBS 12	Oncoplastic breast surgeon	F	<5	50–200
OPBS 13	Oncoplastic breast surgeon	F	>10	25–50
OPBS 14	Oncoplastic breast surgeon	F	5–10	11–20
OPBS 15	Oncoplastic breast surgeon	F	<5	11–20
OPBS 16	Oncoplastic breast surgeon	M	5–10	50–100
OPBS 17	Oncoplastic breast surgeon	M	>10	50–100
OPBS 18	Oncoplastic breast surgeon	M	>10	25–50
OPBS 19	Oncoplastic breast surgeon	M	Trainee	> 100
OPBS 20	Oncoplastic breast surgeon	M	Trainee	50–100
OPBS 21	Oncoplastic breast surgeon	F	Trainee	50–100
OPBS 22	Oncoplastic breast surgeon	F	Trainee	25–50
OPBS 23	Oncoplastic breast surgeon	F	5–10	50–100
OPBS 24	Oncoplastic breast surgeon	M	>10	50–100
OPBS 25	Oncoplastic breast surgeon	F	5–10	11–20
OPBS 26	Oncoplastic breast surgeon	F	>10	50–100
OPBS 27	Oncoplastic breast surgeon	M	<5	25–50
Plastic surgeon 1	Plastic surgeon	F	5–10	50–100
Plastic surgeon 2	Plastic surgeon	F	<5	50–100
Specialist nurse 1	Breast CNS	F	Unknown	25–50
Specialist nurse 2	Breast CNS	F	Unknown	25–50

The interviews focused on the feasibility and acceptability of RCTs in IBBR and the key themes relating to this are presented below. Details of participant gender, speciality and response to the survey question, “Is an RCT in IBBR possible?” (yes, no, unsure) are provided following each quotation to provide the reader with a contextual detail. Details relating to specific aspects of study design (e.g. mesh selection or approach to concomitant interventions) will be reported elsewhere.

### The need for further evidence in IBBR

The need for more evidence to support the practice of implant-based surgery was consistently highlighted by clinicians. This manifested itself in the way surgeons’ felt that crucial questions remained unanswered.

*Fundamentally, there is a question where we don’t know which [implant position] is better and which is worse, and we don’t know which ADM [surgical mesh] is better or which is worse. It would be great to know that. – OPBS 19 (M, trainee, RCT possible)*

Participants also almost universally identified RCTs as the study design that provided the highest levels of evidence.

*But we always need good quality evidence, and good quality evidence comes from randomised trials. – OPBS 1 (F, >10 yrs, RCT possible)*

Despite recognising the need for more evidence and an apparent understanding that RCTs provide the highest quality data, opinion was divided regarding the feasibility of a large-scale RCT in IBBR. Consistent with the survey findings, most respondents felt that a study of this type was needed and was achievable, even whilst highlighting certain challenges that may make conduct more difficult. In contrast, fewer participants were opposed to the idea of an implant reconstruction trial, many of whom came from centres performing high numbers of procedures annually.

The opposition to RCTs and potential challenges to the successful conduct of a future trial in IBBR centred around three common and interlinked themes. These were (i) partial appreciation of the value of RCTs; (ii) clinician and patient equipoise and (iii) issues relating to inherent surgical culture.

### Partial appreciation of the value of RCTs

Despite initially acknowledging that RCTs provide the highest levels of evidence, on further discussion it became apparent that the methodological value of RCT design and robustness of results was not always fully appreciated by those opposed to RCTs in IBBR. It appeared that the issues with bias inherent in non-randomised studies were not fully understood or appreciated by all clinicians. Often the need for further research was identified, but it was felt that the same level of evidence could be achieved using alternative, non-randomised study designs including clinical audit.

*Do we really, really need an RCT? I think we really, really need good data collection and to share our data. I think we all should be open for that. None of us need to reinvent the wheel. There’s lots of information out there. – OPBS 7 (F, >10 yrs, RCT not possible)*

*I think prospective audit is going to give you enough evidence, as long as it is properly audited – OPBS 24 (M, >10 yrs, RCT not possible)*

The value of RCT design in the context of surgery was sometimes felt to be limited due to the nature of surgery as a handcraft discipline. The complexity of the intervention was perceived to introduce a number of variables that would limit the usefulness of any trial results. The concept of pragmatic trial design was often not appreciated or apparently not understood.

*I do understand the value of it and the order of the hierarchy of the evidence, but I think, for surgeons particularly, it’s a handcraft discipline. It’s not like radiotherapy or it’s not like oncology, where you’re delivering a defined intervention. It’s a handcraft and where you do handcraft, the variables come into play. If, at the end of the day, the variables are so significant then any intelligent researcher would be asking the question, “What is the value? What are we trying to achieve here?” – OPBS 20 (M, trainee, RCT not possible)*

Some surgeons struggled with the concept of applying results from a robust pragmatic trial to an individual patient in clinical practice. Surgery was described as an art as well as a science, and there was a feeling that trial results may not be relevant to individual patients.

*In terms of outcomes and things, to my knowledge there are no randomised controlled trials on outcomes, but part of me does think that a lot of this is an art as well as a science. So, I think you can do trials. but I think also it's very much down to the individual patients and their skin quality. – OPBS 21 (F, trainee, RCT possible)*

The idea of randomisation itself drew concern from some professionals. One professional felt from experience that randomisation would hinder recruitment of possible patients. They described patients interpreting randomisation as a computer deciding their treatment and it was felt this negatively biased them against participating in the trial.

*The ladies love the idea of the trial, but they don’t want a computer to make that decision for them, so recruitment for [another breast surgery trial] has been slow. – OPBS 4 (F, 5–10 yrs, RCT possible)*

Other concerns with regards to randomisation were more logistical. The timing of randomisation was highlighted as a possible issue as the discrepancy in operation length would affect list planning.

*But obviously the only difficulty with sub- and pre-pec is would it take much longer in terms of operative time? In which case, you've got to ask the question of when do you randomise, because it may influence the planning of lists and things. - OPBS 21 (F, trainee, RCT possible)*

### (Lack of) clinician and patient equipoise

Equipoise is fundamental to the design and conduct of clinical trials [33] but within IBBR, issues related to a lack equipoise on many levels emerged as a significant barrier to successful trial conduct.

*Yes, I think it’s very difficult to get randomised data. I think it’ll be very difficult to do a randomised controlled trial ... Because of the element of surgeon preference and patient choice. – OPBS 9 (F, <5 yrs, unsure about future RCT)*

A lack of surgeon equipoise was most commonly seen among clinicians from high-volume centres, with more personal experience of the newer surgical techniques.

*I don’t see a reason to go back and do a pre-pectoral on a randomised controlled study. I wouldn’t be able to explain that to my patient, “Well they are equal,” or, “We don’t know whether they are equal,” because I know that it’s not equal. I know pre-pectoral is a lot better. – OPBS 16 (M, 5–10 yrs, RCT not possible)*

Other surgeons highlighted the disparity between community and individual equipoise. They described the lack of good-quality evidence to support specific interventions, but expressed personal preferences towards specific treatment options when deciding the management of individual patients.

*This is all anecdote and I know it’s anecdote, but it’s really difficult if you have that person sitting in front of you. I completely understand that and that is one of the main difficulties isn’t it, that even if it’s anecdotal, you still develop opinions and that sort of stuff? – OPBS 13 (F, >10 yrs, RCT not possible)*

This was commonly the result of what surgeons perceived to be the ‘obvious’ benefits of the newer procedure. Surgeons often demonstrated a lack of equipoise regarding implant position as they felt confident that specific procedures were more beneficial in certain patients. They felt this would make randomisation difficult.

*So, I think there are clear differences between the two, in terms of what I perceive to be the benefits of one over the other. For that reason, yes, I suppose I wouldn’t tell my patients that I was in complete equipoise between the two techniques. In which case, they might feel that randomisation wasn’t for me and they were unfair to consider. – OPBS 9 (F, <5 yrs, unsure about future RCT)*

As a result, surgeons’ often felt that only a very small subset of patients would be suitable for the trial, with genuine equipoise present.

*I think for certain cases, they could be randomised but I think the majority of patients, there are factors that would lead you to go one way or another. So, it would be tricky. – OPBS 14 (F, 5–10 yrs, RCT not possible)*

Surgeons also noted that patients’ preference must be accounted for, and they must be in equipoise for recruitment into randomised trials.

*There will be a group where you could do either, and you'd probably get good results from either, but you’ve also then got to factor in the patient’s preference. It’s not just the surgeon who’s got to have equipoise, it’s the patient. – OPBS 26 (F, >10 yrs, RCT not possible)*

The importance of patient choice in the decision-making process was seen by surgeons as a barrier to involvement in an RCT. Trials were only felt to be acceptable if patients could choose their treatment arm, negating the methodological value of randomisation in terms of minimising selection bias and further highlighting a lack of understanding of RCTs.

*So, I suppose my thoughts about the trial is that I would be happy to recruit a patient to either arm, on the basis that in essence they select which arm they go into. So, I would do either but it would be a patient choice as to which arm they went into. – OPBS 19 (M, trainee, RCT possible)*

In contrast, breast care nurses along with some surgeons highlighted that offering trials was crucial in enabling patients to make a fully informed choice regarding their treatment from the full range of options available.

*I think what's important is that patients know the options. Then if they choose to take them or not, at least they know those options are there. If anybody is eligible for a trial, we always offer that, even if the patient just says, “No, thank you. I don't even want to really know about it. I want to just focus on what is the standard pathway.” Then that's fine, but it's about knowing the options. – Specialist nurse 1 (F, n/a, RCT possible)*

*If you're not offering, you are depriving patients’ choice. – OPBS 23 (F, 5–10 yrs, RCT possible)*

Some clinicians did not appreciate the difference between their role in clinical practice and as a recruiter to an RCT. Trial recruitment was seen to be difficult as they tended to highlight the pros and cons of different treatment choices and then patients formed a preference based on this.

*When you start talking to them about the differences between pre-pectoral and sub-pectoral and say, well this is much less painful, but you might see some rippling, they go, oh well I’ll go for that. – OPBS 15 (F, <5 yrs, RCT not possible)*

Clinicians, who were often senior with more experience of reconstruction, also reported providing a steer to patients who were preoccupied with their cancer diagnosis. Highlighting that these patients did not have pre-formed ideas about treatment, but were instead guided by their clinician.

*They don’t come with preformed ideas about what they want. Not at all ... They’re so wrapped up in their cancer or DCIS [ductal carcinoma in situ] diagnosis that it’s often the talk about reconstruction is secondary, so we very much guide people. – OPBS 2 (F, 5–10 yrs, RCT possible)*

### Inherent surgical culture

A significant proportion of the opposition to RCTs in IBBR seemed to stem from an underlying surgical culture that is unfamiliar with the concept of trials and evidence-based practice.

It became apparent that much of what guides surgical practice is personal, lived experience rather than high-quality evidence of clinical effectiveness.

*I think the sort of whole surgical mind-set is that during your training you see other people doing different things and you work out what you think is the best thing for whatever reason, and then have a kind of dogged determination to stick to your approach, whatever it is, without it really being evidence based or tested or audited in a kind of multicentre way. What works in your hands, works in your hands – Plastic surgeon 1 (F, 5–10 yrs, RCT possible)*

This reliance on personal experience was seen as a barrier to trial participation, as surgeons would be apprehensive about changing the surgical technique they are accustomed to and worked in their hands.

*That’s an interesting one. Probably not, to be honest. I think you’ll find a lot of resistance from people doing what they’re used to doing. – OPBS 3 (F, <5 yrs, RCT not possible)*

One surgeon felt that prior clinical experience was a major factor in this. More experienced clinicians were felt to be more likely to rely on their extensive clinical experience and would not be in sufficient equipoise to recruit.

*My comments would be yes, it would be possible [to recruit to trials] and there are certainly plenty of surgeons out there that aren’t established in one or the other, so you probably would find people who are still relatively naïve that would be able to recruit, but I think some of the rest of us that are a bit longer in the tooth might find it a bit more challenging. – OPBS 13 (F, >10 yrs, RCT not possible)*

Another barrier to trial participation raised by a number of surgeons was the logistical issue of recruiting patients to trials when facing patients already burdened by a new cancer diagnosis. Surgeons described having difficulty with recruiting as a result of the treatment deadlines and the burden of information that needed to be conveyed to the patient.

*I think we need to remember these patients, we’re bound by 31 and 62 day targets and we’re throwing a whole load of information at them. We give them a cancer diagnosis and we give them a lot of information about their reconstruction … I think adding in a trial on top of that can be really, really confusing for the patients. – OPBS 7 (F, >10 yrs, RCT not possible)*

Even senior surgeons acknowledged their discomfort when discussing complex surgical trials with patients. This in itself was seen as a barrier in some cases.

*I’m not sure an RCT will be easy. The ones we’ve tried to do with regard to [another type of breast reconstruction], it’s very, very difficult. I’ve sat and tried to have a preliminary discussion with patients prior to [the] trial and I was confusing myself, to be honest. I think an RCT would be difficult. I’m not saying it would be impossible, but I think it would be very difficult. – OPBS 7 (F, >10 yrs, RCT not possible)*

However, this was also seen as a key communication skill and was acknowledged as something that all surgeons should learn and possess.

*I think it's all about how you explain it to the patient, but it's absolutely achievable. If people say you can't do it, it's they can't do it. They're failing to do it. It's a communication skill that everyone should have and should learn. – OPBS 23 (F, 5–10 yrs, RCT possible)*

Only one surgeon openly acknowledged the possible discrepancy in their role as both a clinician and a trialist. It was apparent that, despite realising the need for an increased evidence base, there was some reluctance to recruit to clinical trials for fear of less positive clinical results.

*Losing one [an implant], that’s massive. So, it’s a bit scary, but that doesn’t mean I don’t think getting that evidence is valuable. I appreciate it is unacceptable for me to say, “I want that evidence,” but I don’t want to lose my results. Someone else can do the dirty work. (Laughter) – OPBS 4 (F, 5–10 yrs, RCT possible)*

## Discussion

This study provides an overview of the attitudes of potential recruiters towards a future RCT in IBBR and highlights the possible barriers to successful trial conduct. Although the majority of participants identified the need for better evidence to support the practice of IBBR, fewer were supportive of an RCT to answer these questions. Three key themes emerged as underpinning much of the opposition to RCT conduct: a limited appreciation of the methodological value of RCTs, in particular the importance of randomisation in minimising bias and the concept of pragmatic study design; issues related to clinician and patient equipoise, and a surgical culture that is less familiar with the concept of RCTs and evidence-based practice than other medical specialties.

Previous qualitative work in reconstructive breast surgery [22] emphasised the need for a change in surgical research culture and the education of surgeons themselves to improve recruitment to surgical trials. They cited a lack of familiarity with, and understanding of, the concepts of RCT methodology and a poor appreciation of the hierarchy of evidence as obstacles to successful conduct of an RCT in reconstructive breast surgery. Whilst these themes have re-emerged within this work, it appears that a greater proportion of surgeons are now confident in the benefits of and need for RCTs to provide high-quality evidence in order to guide clinical practice. Surgeons also felt comparatively more comfortable with the implementation and conduct of randomised trials within their clinical practice. Both the advent and growth of surgical trainee research collaboratives [34] and the establishment of Surgical Trials Initiative by the Royal College of Surgeons of England [35] in recent years may be partly responsible for the increase in engagement and capacity for high-quality research within surgery.

The potential barriers to recruitment identified in this study are not unique to trials in breast reconstruction [36, 37] and development of strategies to improve recruitment to RCTs is a research priority [38]. Evidence for the effectiveness of existing approaches has been summarised in several recent systematic reviews and has shown to be limited [15, 36, 37, 39–41]. Qualitative research within trials to explore barriers to recruitment, however, is likely to be an effective strategy for allowing recruitment challenges to be understood and overcome [37].

Indeed, although the challenges highlighted in this study are perceived as barriers to successful trial conduct by study participants, previous qualitative research has demonstrated that these issues can be explored and addressed resulting in successful trials in areas that had previously been deemed impossible [42]. Patient preference was often identified as a barrier to trial recruitment, an issue which has previously been reported to be particularly problematic within breast reconstruction [22, 24]. Despite this, earlier work has demonstrated that by exploring these preferences, enabling any concerns or misconceptions to be unearthed and addressed, the majority of patients were open to other treatment choices and consented to randomisation [43]. Participants also perceived that patients would be reluctant to have their treatment ‘decided by a computer’. Whilst this is commonly stated when explaining randomisation, it has been shown that computer-agency descriptions can impede recruitment, suggesting that it is the communication of the process that can cause difficulties [44]. Lack of surgeon equipoise emerged as a major barrier to participation in a future trial even though, in contrast to previous work [22], participants appeared to be aware that there was limited evidence to support their views. The discomfort expressed by many surgeons in the study about equipoise and ‘knowing’ what treatment may be best for each individual patient is not uncommon in RCTs [19] and undermining or contradicting the concept of equipoise has been shown to be a barrier to recruitment to trials [45].

Improved understanding of the fragility and complexity of trial recruitment has led to the development of the QuinteT Recruitment Intervention (QRI) [46]. The QRI is a flexible, tailored intervention embedded in challenging to recruit to RCTs that aims to identify and address recruitment difficulties in real time. The intervention has now been used in over 30 trials and there is increasing evidence to support its value [47–50]. As many of the challenges centre on communication of key trial concepts such as equipoise and randomisation that are common across different contexts, recruiter training workshops have also been developed which have been shown to increase professionals’ confidence in discussing RCTs with patients, raise awareness of the hidden challenges of RCTs and have a perceived positive impact on recruitment practice [51] It is likely that embedding a QRI and recruitment training into a future trial would allow many of the perceived challenges to be effectively addressed and overcome.

This study has several possible limitations. First, it included a pragmatic sample of clinicians who engaged in interviews. All clinicians had previously completed the RCT acceptability survey and had indicated that they would be willing to discuss their views in a telephone interview, thereby biasing towards a sample who are already actively engaged in surgical research. However, this likely represents the population of clinicians who will engage with future RCTs in IBBR and therefore may be entirely appropriate. Plastic surgeons and clinical nurse specialists were comparatively under-represented in the sample. Plastic surgeons perform IBBR less commonly than oncoplastic breast surgeons and are often based in different hospitals or clinics. From a surgeon perspective therefore, study participants largely reflected potential trial recruiters. The lack of CNS involvement, however, is a potential concern as previous work has demonstrated that the whole team needs to be engaged and invested in the trial if it is to be successful [52, 53]. This is particularly important in breast reconstruction studies as specialist nurses play an integral role in patient decision-making. Multiple attempts were made to engage this group, but these were unsuccessful. It will be essential for any recruitment intervention within a future trial to fully involve this vital professional group. It is possible that the researcher (GD, medically-qualified male) shaped the study by unknowingly influencing interview responses and data analysis given his medical background. However, he has no experience with breast reconstruction and had not been involved in previous phases of the iBRA study, and it is possible that clinicians may have been more open in their answers and comfortable with the discussion knowing they were talking to another clinician. Throughout the study we attempted to ensure that multiple different perspectives were reported to avoid representation of only one group and that there was agreement within the study team regarding coding and emerging findings, To allow readers to appraise the data and the conclusions drawn from them, a detailed description of the data collection and analysis process have been provided, along with a number of quotations to illustrate the themes discussed.

This study suggests that the reconstructive community is engaged in the need for better evidence to support the future practice of IBBR. The majority interviewed here would be supportive of a future trial and many of the barriers to RCTs proposed in this study are well-established in surgical trials and could potentially be effectively addressed by recruiter education and training. These findings, in combination with the results from the earlier phases of the iBRA study [9], which suggest that different approaches to IBBR may be equivalent in a non-randomised study, strongly support the need for an RCT in IBBR. The next step will be to design and deliver the Best-BRA study, an external pilot RCT with an embedded QuinteT Recruitment Intervention comparing two different approaches to implant placement that will establish whether an RCT in IBBR is possible. Ongoing education of surgeons regarding the need for high-quality evidence will also be important and the Association of Surgery iBRA-NET initiative, which aims to engage surgeons in the concept of ‘no innovation without evaluation’, will be an key means of achieving this and addressing some of the cultural issues identified in this study.

## Conclusion

There is a need for well-designed large-scale RCTs in IBBR but barriers to their acceptability are evident. The perceived barriers to a future RCT identified in this study, however, are not insurmountable and could be successfully overcome through recruiter education and training. This may represent an opportunity, not only to establish the evidence base for IBBR, but also to improve engagement in RCTs in breast surgery in general to ultimately improve outcomes for patients.

## Supplementary information

**Additional file 1.**

**Additional file 2.**

## Data Availability

The data from this study are not publicly available due to them containing information that could compromise research participant privacy/consent.
