# Supplementary material for: Perceived barriers to randomised controlled trials in breast reconstruction: obstacle to trial initiation or opportunity to resolve? A qualitative study
Source: Trials. 2020 Apr 6;21:316. doi: 10.1186/s13063-020-4227-1 (PMC7132957; doi:10.1186/s13063-020-4227-1)
Supplement: Supplementary file 1 — Additional file 1. [file 13063_2020_4227_MOESM1_ESM.pdf]

iBRA Randomisation Acceptability Survey

V2: 06/02/2018. FREC ID:61501

Invitation to complete the iBRA Randomisation Acceptability Survey

Dear iBRA collaborators and colleagues,

Many thanks for your help with the iBRA study. iBRA has recruited over 2000 patients from over 50 centres in the UK making it the largest prospective evaluation of implant-based breast reconstruction (IBBR) in the world.

What is the purpose of the study?

iBRA has demonstrated significant variation in the practice and outcomes of IBBR in the UK but data suggest that the short-term outcomes of reconstruction performed with different types of mesh (e.g. biological and synthetic meshes) may be similar at 3 months. Outcomes of prepectoral and subpectoral reconstruction may also be similar although the numbers of patients having pre-pectoral reconstruction is relatively small. There is therefore a need for further well-designed research to determine best practice in IBBR and improve outcomes for patients.

Randomised clinical trials (RCTs) provide the best quality evidence but previous RCTs in breast reconstruction have been unsuccessful as they did not involve reconstructing surgeons in designing a study that answered the most important questions in the most acceptable way.

The iBRA Steering Group would like to invite you to complete the following survey to help inform the design a large pragmatic RCT to determine which approaches to IBBR give the best outcomes for patients and are the most cost-effective for the NHS.

We would like to know where you feel the areas of uncertainty are in mesh-assisted implant reconstruction; what trial designs you think would be acceptable and you would be happy to randomise patients into; what outcomes we should use and finally how pragmatic the trial could be with regard to implant and mesh use and concomitant interventions such as use of drains and antibiotics.

Why have I been invited?

You have been invited to participate as you offer patients IBBR as a treatment option or are involved in counselling patients considering surgery.

You and/or your unit do not need to have participated in the earlier phases of the iBRA study to complete this survey. We would like to include the views of as many breast and plastic surgeons and clinical nurse specialists as possible to inform a future trial.

What will participation involve?

The survey should take no more than 20 minutes to complete.

We would also like to interview a sample of surgeons and specialist nurses completing the survey to explore their views in more detail.

Taking part in the interview study is completely optional and you can complete the survey only if you choose.

If you would be willing to be interviewed, please complete this section of the survey and we will contact you with further details.

We would be very grateful if you could also circulate the link to other colleagues and specialist nurses involved in implant-based breast reconstruction.

Do I have to take part?

Taking part is voluntary.

You can complete the survey only if you choose without agreeing to be interviewed.

Anonymised data will be made available on request to other researchers at the end of the study.

If you do not provide contact details, your responses will be completely anonymous, and it will not be possible to withdraw your data from the study.

If you have provided contact details, it is possible to withdraw your data after completing the survey if you wish.

Who is organising and funding the research?

This study has been reviewed and given a favourable opinion by the University of Bristol Faculty of Health Sciences Research Ethics Committee (reference: 61501) and is funded by the National Institute for Health Research (NIHR) Research for Patient Benefit Programme.

If you would like to take part, please click on the link.

If you have any concerns or would like further information

If you have any questions or queries about the study or would like to read a full copy of the study protocol, please do not hesitate to contact Miss Shelley Potter via e-mail (shelley.potter@bristol.ac.uk) or telephone (0117 9287218).

If you have a formal complaint or any concerns about this research, you may contact the University of Bristol’s Research Governance team (research-governance@bris.ac.uk).

Many thanks in advance for your help in designing this important study

Best wishes

Miss Shelley Potter and Professor Chris Holcombe

On behalf of the iBRA Steering Group

PART 1 - DEMOGRAPHIC DETAILS AND PRACTICE OF IMPLANT-BASED BREAST RECONSTRUCTION

1a Are you a

◯ Consultant Breast Surgeon
◯ Consultant Plastic Surgeon
◯ Senior breast trainee (ST7/8 or above)
◯ Senior plastics trainee (ST7/8 or above)
◯ Speciality doctor/Associate Specialist
◯ Breast care specialist nurse
◯ Breast reconstruction specialist nurse
◯ Other

Please give details ________________________________

1b Is immediate implant-based breast reconstruction offered in your unit?

◯ Yes, I am a surgeon and I perform implant-based reconstruction
◯ Yes, I am a specialist nurse and I counsel patients considering implant reconstruction
◯ No, implant reconstruction is not offered in my unit

1c Approximately how many immediate implant-based breast reconstructions overall were performed in your unit last year?

◯ Less than 5 ◯ 5-10 ◯ 11-20 ◯ 25-50 ◯ 50-100 ◯ more than 100

1d Did your Unit recruit patients to the iBRA (implant Breast Reconstruction evAluation) Study?

◯ Yes ◯ No ◯ Not sure

1e Which of the following techniques are routinely perform or are routinely offered in your unit? (Please tick all that apply)

☐ Standard 2 stage subpectoral reconstruction without mesh
☐ Subpectoral implant reconstruction with dermal sling
☐ Subpectoral implant reconstruction with biological mesh (e.g ADM such as Strattice or SurgiMend)
☐ Subpectoral implant reconstruction with synthetic mesh (e.g. TiLOOP, TiGR)
☐ Prepectoral reconstruction with dermal sling +/- mesh
☐ Prepectoral reconstruction with biological mesh (e.g ADM such as BRAXON)
☐ Prepectoral reconstruction with synthetic mesh (e.g TiLOOP, TiGR)
☐ Other

If other, please give details

____

1f. What products/techniques have been used in your Unit in the PAST YEAR? (please tick all that apply)?

	SUBPECTORAL	PREPECTORAL
Biological (e.g Strattice, BRAXON)	☐	☐
Synthetic (e.g TiLOOP)	☐	☐

PART 2 - UNCERTAINTIES IN THE CURRENT PRACTICE OF IMMEDIATE IMPLANT-BASED BREAST RECONSTRUCTION

We would like to design a randomised clinical trial that healthcare professionals feel addresses an important clinical question and into which surgeons would feel confident to recruit eligible patients.

2a	Do you feel there is uncertainty regarding the best practice of immediate implant based breast reconstruction?	◯ Yes ◯ No ◯ Unsure

2b. Do you feel there is uncertainty in the following areas?

		Yes	No	Unsure
i	The use of MESH compared with NO MESH in implant-based breast reconstruction?	○	○	○
ii	The use of BIOLOGICAL (e.g ADM) versus SYNTHETIC mesh (e.g TiLOOP) in SUBPECTORAL implant-based breast reconstruction	○	○	○
iii	The use of DIFFERENT TYPES OF BIOLOGICAL MESH in SUBPECTORAL implant reconstruction (e.g Strattice vs SurgiMend)	○	○	○
iv	The use of DIFFERENT TYPES OF SYNTHETIC MESH in SUBPECTORAL implant reconstruction (e.g TiLOOP vs TiGR)	○	○	○
v	The best POSITION of the implant (SUBPECTORAL or PREPECTORAL) in implant-based breast reconstruction	○	○	○
vi	The BIOLOGICAL vs SYNTHETIC MESH in PREPECTORAL implant breast reconstruction	○	○	○
vii	The use of FIXED VOLUME vs ADJUSTABLE IMPLANTS in implant-based breast reconstruction	○	○	○
viii	The best way to reduce infection/implant loss in implant-based breast reconstruction	○	○	○
ix	The most COST-EFFECTIVE approach to implant-based breast reconstruction	○	○	○

2c Do you think there are any other areas of uncertainty in immediate implant-based breast reconstruction? ○ Yes ○ No ○ Unsure

Why do you think this?

2d Do you think a randomised clinical trial is NEEDED in implant-based breast reconstruction? ○ Yes ○ No ○ Unsure

Why do you think this?

2e Do you think a randomised clinical trial is POSSIBLE in implant-based breast reconstruction (please tick as many as apply)?

☐ Yes
☐ Possibly if the design is acceptable
☐ No, because of surgeon preference
☐ No, because of patient preference
☐ No, for another reason

Please give details of other reasons why an RCT would not be possible ________________________

Why do you think this?

2f Do you feel that another type of study would be more appropriate? ◯ Yes ◯ No ◯ Unsure

What type of study? ________________________

PART 3 - TRIAL DESIGNS AND OUTCOMES

COMPARATOR ARMS FOR AN IMPLANT RECONSTRUCTION TRIAL

3a. Which of the following randomised trial designs, if any, would you consider acceptable (i.e if open would you consider recruiting eligible patients into)

		Yes	No	Unsure
i	SUBPECTORAL implant reconstruction with MESH vs. PREPECTORAL implant reconstruction with MESH	◯	◯	◯
ii	SUBPECTORAL implant reconstruction with either a BIOLOGICAL MESH (e.g Strattice or SurgiMend) or a SYNTHETIC MESH (e.g TiLOOP or TiGR)?	◯	◯	◯
iii	PREPECTORAL implant reconstruction with either a BIOLOGICAL MESH (e.g Strattice or SurgiMend) or a SYNTHETIC MESH (e.g TiLOOP or TiGR)?	◯	◯	◯
iv	SUBPECTORAL reconstruction with MESH using FIXED VOLUME vs ADJUSTABLE IMPLANTS	◯	◯	◯
v	SUBPECTORAL implant reconstruction with 2 DIFFERENT TYPES of BIOLOGICAL MESH (e.g Strattice vs. SurgiMend)	◯	◯	◯
vi	SUBPECTORAL implant reconstruction with 2 DIFFERENT TYPES of SYNTHETIC MESH (e.g TiLOOP vs TiGR)	◯	◯	◯

3b Are there other RCT designs (other than the ones above) that you would consider acceptable ◯ Yes ◯ No ◯ Unsure

If yes, please give details

3c. Please RANK the proposed RCTs from 1 to 6 in order of feasibility from 1 (MOST feasible) to 6 (LEAST feasible)

	1 - MOST feasible RCT design	2	3	4	5	6 - LEAST feasible RCT design
SUBPECTORAL implant reconstruction with MESH vs. PREPECTORAL implant reconstruction with MESH	○	○	○	○	○	○
SUBPECTORAL implant reconstruction with either a BIOLOGICAL MESH (e.g Strattice or SurgiMend) or a SYNTHETIC MESH (e.g TiLOOP or TiGR)?	○	○	○	○	○	○
PREPECTORAL implant reconstruction with either a BIOLOGICAL MESH (e.g Strattice or SurgiMend) or a SYNTHETIC MESH (e.g TiLOOP or TiGR)?	○	○	○	○	○	○
SUBPECTORAL reconstruction with MESH using FIXED VOLUME vs ADJUSTABLE IMPLANTS	○	○	○	○	○	○
SUBPECTORAL implant reconstruction with 2 DIFFERENT TYPES of BIOLOGICAL MESH (e.g Strattice vs. SurgiMend)	○	○	○	○	○	○
SUBPECTORAL implant reconstruction with 2 DIFFERENT TYPES of SYNTHETIC MESH (e.g TiLOOP vs TiGR)	○	○	○	○	○	○

OUTCOME SELECTION

3c Please rank which of the following you would consider the most important outcome to assess in an IBBR RCT from 1 (MOST important) to 9 (LEAST important)

	1 - MOST IMPORTANT	2	3	4	5	6	7	8	9 - LEAST IMPORTANT
Implant loss	○	○	○	○	○	○	○	○	○
Infection	○	○	○	○	○	○	○	○	○
Unplanned re-operation for complications/unacceptable cosmetic outcome	○	○	○	○	○	○	○	○	○
Unplanned readmission for complications	○	○	○	○	○	○	○	○	○
Pain	○	○	○	○	○	○	○	○	○
Patient satisfaction using a validated patient reported outcome measure	○	○	○	○	○	○	○	○	○
Panel assessed cosmetic outcome	○	○	○	○	○	○	○	○	○
The cost-effectiveness of the interventions	○	○	○	○	○	○	○	○	○
Other	○	○	○	○	○	○	○	○	○

Please give details of any OTHER outcomes that would be the most important to measure in a future RCT

____

3d WHEN should the primary outcome for the trial be assessed?

○ 30 days ○ 3 months ○ 6 months ○ 12 months ○ 5 years ○ More than 5 years
○ Other

If other, please give details

____

PART 4 - PARTICIPATION IN TELEPHONE INTERVIEW QUALITATIVE STUDY

We would like to interview a sample of surgeons and specialist nurses who have completed the survey to explore their views in more detail.

Interviews will be conducted by TELEPHONE and are likely to take NO MORE THAN 30 MINUTES.

If you are potentially interested in participating, please enter your e-mail address and we will contact you within the next month with more details.

4a 4a. I am happy to be contacted to consider participating in a brief telephone interview about RCTs in implant-based breast reconstruction

◯ Yes ◯ No

If yes; please provide a contact e-mail address

____
