# Supplementary material for: Perceived barriers to randomised controlled trials in breast reconstruction: obstacle to trial initiation or opportunity to resolve? A qualitative study
Source: Trials. 2020 Apr 6;21:316. doi: 10.1186/s13063-020-4227-1 (PMC7132957; doi:10.1186/s13063-020-4227-1)
Supplement: Supplementary file 2 — Additional file 2. [file 13063_2020_4227_MOESM2_ESM.pdf]

Topic guide for iBRA phase 3 qualitative interviews interviews with surgeons and specialist nurses involved in performing or counselling patients undergoing implant-based breast reconstruction

Introduction:

- Thank you for taking time
- Want to explore views about RCTs in implant-based breast reconstruction No right or wrong answers, can stop the interview at any time
- Take verbal consent
- Remind them that the interviews are being audio-recorded, assure confidentiality

Can you tell me about yourself?

- What is your current position, role and level of experience in the field
- What types of implant reconstruction are performed in your unit (pre-pectoral/subpectoral; different types of meshes)
    - Why is this/why do you think this is
    - Evidence for this/participation in earlier phases of iBRA

i. Study designs and comparators

As you know, one of the aims of iBRA was to determine the feasibility of undertaking a RCT in IBBR so I'm interested in hearing your views around this.

- Do you think it's possible to undertake an RCT in IBBR? (explore views)
- Would another type of study design be better? (explore views)
- What do you think your colleagues (surgeons and nurses) might think?
- How comfortable would you feel in inviting patients to an RCT in IBBR? (explore views)
- Do you think patients would be likely to participate? Why?
- What would make a trial possible?
- What would make a trial difficult?
- Do you think there could be sufficient equipoise amongst surgeons and nurses to make an RCT feasible?
- Where do you think there is equipoise *(different types of mesh; pre-pec/subpectoral implants; different types of implants)*
- What studies would you be happy to recruit patients in to (and why)?
- Which would you ***not*** be happy to recruit into and why?
- What do you think the challenges of a potential RCT would be?
- How do you think these could be overcome?
- Explore any specific issues raised in survey

ii. Degrees of pragmatism and attitudes to concomitant interventions

We are keen to ensure that any future trial is acceptable to surgeons performing IBBR and is also pragmatic (reflects the real world).

What are your thoughts? Should all aspects of the procedure be standardised or should surgeons be allowed to decide for themselves about things like drains and antibiotics?

- What things do you think should be standardised, if any?
- What things can be left to individual surgeons to decide? *(specifically ask about infection prevention bundles incl. antibiotics; types of mesh; drains; follow up etc)*

- How would you feel if the trial mandated an SSI bundle/specific type of mesh etc?
- Explore any specific issues raised in survey responses

iii. <u>Outcome selection</u>

What outcomes do you think we should measure in the trial? Who is best place to determine these?

- Primary outcomes?
- Secondary outcomes?
- How do you think they should be assessed?
- When do they think they should be assessed?
- Explore any specific issues raised in the survey

Ending the interview

- Is there anything else that I haven't mentioned or that you want to add?

Thank for time, reiterate confidentiality, summarise key points
